# Knowledge and Management of Halitosis in France and Lebanon: A Questionnaire-Based Study

**DOI:** 10.3390/jcm10030502

**Published:** 2021-02-01

**Authors:** Laetitia Harmouche, Yves Reingewirtz, Nicolas Tuzin, François Lefebvre, Jean-Luc Davideau, Olivier Huck

**Affiliations:** 1 Department of Periodontology, Faculté de Chirurgie Dentaire, Université de Strasbourg, 67000 Strasbourg, France; laetitia.harmouche@gmail.com (L.H.); jldcabfra@wanadoo.fr (J.-L.D.); 2 Periodontology, Pôle de Médecine et Chirurgie Bucco-Dentaire, Hôpitaux Universitaires de Strasbourg, 67000 Strasbourg, France; yves.reingewirtz@orange.fr; 3 Groupe de Méthodes en Recherche Clinique, Hôpitaux Universitaires de Strasbourg, 67000 Strasbourg, France; nicolas.tuzin@chru-strasbourg.fr (N.T.); francois.lefebvre@chru-strasbourg.fr (F.L.); 4 INSERM 1260 Regenerative Nanomedicine Fédération de Médecine Translationnelle de Strasbourg (FMTS), 67000 Strasbourg, France

**Keywords:** halitosis, oral malodor, oral breath, diagnosis, treatment, questionnaire

## Abstract

Halitosis is a growing issue and its management is highly challenging. The aim of this study was to evaluate the knowledge and treatment strategies used by French (FD) and Lebanese (LD) dentists. A self-administered structured questionnaire was sent to FD and LD comprising questions about professional characteristics, management, and treatment of halitosis, patients’ referral, and halitosis-related knowledge. A multivariate analysis was conducted to determine differences between FD and LD and to identify parameters that could influence dentists’ management of halitosis. The questionnaire was filled out by 156 FD and 257 LD. Among them, 78.8% of FD and 68.9% of LD were confronted with halitosis management, while only a few routinely asked their patients about halitosis (16% FD, 13.2% LD). Regarding anamnesis, oral hygiene habits were more investigated by FD than LD (*p* < 0.05). The overall treatment satisfaction was low with 39.7% of FD and 28.4% of LD considering their treatment effective. Regarding halitosis-related knowledge, extra-oral causes were overestimated in both populations. FD (83.4%) and LD (65.8%) considered their education regarding halitosis as insufficient. This study highlights the need of professional education in both countries, targeting proper diagnosis and treatment strategies of halitosis.

## 1. Introduction

Halitosis is a common disorder as it affects 22 to 50% of the global population [1]. Indeed, a recent meta-analysis estimated the global prevalence of halitosis being around 32% in adolescents and adults [2]. Owing to its substantial social impact, halitosis can affect quality of life, contributing to a significant growth of the market for pharmaceutical products against bad breath. Interestingly, halitosis has also been linked to oral and systemic conditions. Indeed, it was reported that individuals with periodontitis exhibit 3.2 higher odds of oral malodor [3] and a potential role for volatile sulfur compounds (VSC) in the gingivitis-to-periodontitis transition has been suggested [4], emphasizing the importance of halitosis consideration. The worldwide classification of halitosis distinguishes genuine halitosis from psychological halitosis (pseudo-halitosis and halitophobia), the latter representing 5 to 25% of the cases [5,6]. Genuine halitosis is subclassified into physiologic halitosis and pathologic halitosis. Pathologic halitosis can be of intra-oral or extra-oral origins, and it has been demonstrated that the intra-oral causes such as periodontal diseases, tongue coating, and tooth decay are involved in more than 75% of the cases [6]. Therefore, due to its high prevalence and a predominant intra-oral etiology, dentists should play a major role in the management of this condition [7,8].

Over the last few decades, several studies have been conducted to develop new diagnosis and treatment strategies for halitosis [9]. However, it seems that knowledge or information regarding halitosis is lacking not only among the general population, but also among medical and dental specialists [10]. In separate studies performed in China and Belgium, it was observed that 30% of Chinese patients complained about having suffered from halitosis for over 5 years before seeking treatment [10] while almost 33% of Belgian patients had experienced oral malodor for over 5 years before visiting an oral malodor clinic [6]. Interestingly, only a few studies have assessed the knowledge of dental professionals about halitosis [7,8,11]. They highlighted that Swiss dentists were ahead in terms of halitosis management while other dentist populations would benefit from clinical guidelines. Such country-based evaluations are important as the management of halitosis could be influenced by the social and cultural perception of halitosis. For instance, Asians are less knowledgeable about the definition of disease processes than are White adults, but are more concerned about the aesthetic and social consequences of poor oral health [12]. Other studies have been performed in European populations, such as the study by Schemel-Suarez et al. performed with Spanish dentistry students [13]. Indeed, it was observed that self-perception of halitosis was not systematic and cited causes were sparse, including food (33%), multifactorial (food, improper hygiene, respiratory) (18%), digestive (7%), or respiratory (4%). Such observations were also observed in a Saudi population [14]. Nevertheless, different eating, smoking, and drinking habits affect the perception of the stigma associated to halitosis [15]. Thus, the dental professionals should be aware and sensitive to the cultural perceptions of halitosis among racially and culturally diverse populations.

In France, it was observed that 22% of a sample of 4817 subjects reported bad breath [16] while in Lebanon, a culturally different country, self-perception of malodor was reported by 44% males and 54% of females of a sample population of 498 subjects [17]. In both countries, there is a high demand for halitosis management, however, halitosis-related education is limited. Moreover, evaluation of the knowledge and management of halitosis in the dental community is also limited. Therefore, the aim of this study was to evaluate them among dentists of culturally diverse environments. For this purpose, a survey was conducted among French (FD) and Lebanese (LD) dentists to determine their need of halitosis-related professional education.

## 2. Materials and Methods

### 2.1. Study Sample

This study received approval from the Ethics Committee of the Faculty of Medicine, Strasbourg University (CE-2021-5). An online questionnaire was sent to FD and LD via email using Google Forms. The electronic addresses were obtained from several French dental associations (636 email addresses) and from the Lebanese Dental Association (2940 email addresses). Each subject was clearly informed about the aim and content of the survey. The submission of the questionnaire implied their full consent. This study was conducted between January and June 2017.

### 2.2. Questionnaire Design

This study was carried out using self-administered structured questionnaires. The questionnaire comprised 27 questions grouped into 5 domains: (1) dentists’ demographic and professional characteristics, (2) diagnosis of halitosis, (3) treatment of halitosis, (4) referral of patients, and (5) knowledge level of halitosis. Questions included yes/no and multiple-choice questions. All questions had to be answered to submit the questionnaire and approximately twelve minutes were required to complete it. Prior to the study, the questionnaire was pre-tested in France and Lebanon to validate it and then apply the required modifications.

### 2.3. Data Extraction and Statistical Analysis

All data were computerized in a dedicated database. Quantitative variables were described by using position and variability statistics such as mean, variance, minimum, maximum, and quantiles. Qualitative variables were described by using effectives and proportions for each of their modalities. Cumulative proportions were calculated for variables with more than two modalities. The Gaussian distributions of the quantitative variables were evaluated using the Shapiro–Wilk test. The qualitative variables were compared using the Chi-square test if the conditions were met, otherwise the exact Fisher test was employed. Student *t*-test and Mann–Whitney U-tests were used to compare quantitative variables between groups. Linear and logistic multivariate regression were used to consider potential confounding factors (gender, age, and professional experience). A *p*-value < 0.05 was considered statistically significant. In the case of a low number of observations in one category, a model of logistic regression based on Gelman et al. was applied [18]. All analyses were performed using R software under its version 3.0 (R Core Team (2014). R: A language and environment for statistical computing, Vienna, Austria).

## 3. Results

### 3.1. Demographic Characteristics of the French and Lebanese Populations

The response rates of the FD and LD were 24.5% (156/636) and 8.7% (257/2940), respectively (Table 1). Female and male dentists were equally represented and the majority had at least 20 years of professional experience (59% of FD and 38.1% of LD) with most of them having a general practice. Nevertheless, the majority of the surveyed dentists reported to attend professional education sessions more than twice a year (72.4% for FD and 56.8% for LD). The demographic characteristics of the surveyed French and Lebanese dentist populations were significantly different in terms of age (*p* < 0.01), professional experience (*p* < 0.01), type of practice (*p* < 0.01), and specialization (*p* < 0.01). Interestingly, FD attended more professional education courses (*p* < 0.01).

### 3.2. Consideration of Halitosis during Dental Consultation

Despite LD being more frequently faced with patients presenting signs of halitosis than FD (49% vs. 37.2%, respectively) (Table 2), the same proportions of dentists in both countries received patients seeking specific advice to treat this clinical feature (*p* > 0.05). Interestingly, less than 20% of dentists in France (16%) and Lebanon (13.2%) systematically ask their patients about halitosis, while more than 60% claimed to pay systematic attention to this feature (61% for FD and 65.8% for LD). Interestingly, dentists not asking patients about halitosis were mainly concerned about the patient’s eventual discomfort (68.4% for FD and 56% for LD). Multivariate analysis showed that LD were more prone to inform patients about halitosis if they detected it during a check-up (OR = 3.47, *p* < 0.01).

### 3.3. Management of Halitosis

A large majority of dentists declared having already treated halitosis (78.8% for FD and 68.9% for LD) (Table 3). Halitosis treatment is not frequent, as around 70% of the dentists reported to have treated less than 5 patients for halitosis in the last 6 months (66.7% for FD and 78.5% for LD). To determine halitosis etiology, anamnesis focusing on oral hygiene habits appeared to be the most relevant parameter. The anamnesis related to oral hygiene habits was performed more by the FD and when the influence of gender, age and professional experience were considered, it appears that FD investigate oral habits 2.86 (1.18–6.94) more than LD (*p* = 0.02). Accordingly, a full mouth periodontal examination is performed by most of the dentists (64.7% for FD and 54.1% for LD) and only a minority consider the use of specific tools such as organoleptic score measurement or volatile sulfur compounds analysis. Notably, investigation of the patient’s eating habits was performed more by LD (2.22 (1.24–3.98); *p* = 0.007).

To treat halitosis with intra-oral etiology, most dentists provide oral hygiene instructions and perform adequate caries and periodontal treatment. The prescription of specific toothpastes, mouthwashes, and tongue scrapers is not performed systematically. The overall satisfaction of the treatment is very low since only 39.7% of the FD and 28.4% of the LD considered their treatment effective. Indeed, the need of professional education focusing on halitosis appears to be of importance for dentists envisaging appropriate treatment of halitosis (Table 4). Contrary to the observed diagnostic methods, no significant differences in terms of treatment modalities were observed between FD and LD (*p* > 0.05).

Interestingly, only 26.9% of the FD and 38.1% of the LD (*p* < 0.05) have already referred patients with halitosis and, mostly to an ear, nose, and throat (ENT) specialist. After multivariate analysis (gender, age, and professional experience), LD referred their patients 2.02 times (1.17–3.49; *p* = 0.01) more than did FD. The lack of referrals to a halitosis specialist was essentially due to dentists’ lack of knowledge regarding specialists or institutes (Table 4).

### 3.4. Evaluation of Dentists’ Knowledge Related to Halitosis

In both countries, dentists consider their level of education about halitosis provided during their studies as insufficient or non-existent (83.4% for FD and 65.8% for LD) (Table 5). However, only a small number of the dentists participated in continuous educational courses focusing on halitosis (25.6% for FD and 20.6% for LD; *p* = 0.05) due to the lack of availability of such courses/educational programs, especially for LD (62.8%; *p* < 0.01 vs. FD) and also because halitosis management education is not considered a high priority. Interestingly, this lack of education was confirmed as a significant proportion of dentists, in both populations, had misbeliefs regarding halitosis. For instance, only 36.5% of FD and 51.3% of LD were aware of physiological halitosis and a majority (61% of FD and 73.9% of LD) overestimated the involvement of extra-oral causes. Considering their knowledge related to halitosis treatment, only 25% of FD and LD think that referral to a psychologist is important in cases of halitophobia. Interestingly, the sources of knowledge related to halitosis were different between FD and LD (*p* < 0.001). While 57% of the FD acquired their knowledge from scientific journals, the knowledge of LD was largely based on undergraduate education. However, this observation should be correlated with the differences in terms of age and experience between the FD and LD populations, with LD comprising more young professionals than that of FD (*p* < 0.001). When multivariate analysis was conducted considering the influence of gender, age, and professional experience, it was measured that LD were more satisfied by their education toward halitosis than were FD (*p* < 0.001) and more considered themselves as qualified to diagnose and treat halitosis patients (*p* < 0.05), even if they attended continuous education on this specific subject less frequently (*p* < 0.05).

### 3.5. Influence of Demographic Characteristics on Halitosis Management

In both populations, the demographic characteristics and continuous education level were associated with dentist’s halitosis consideration and management. In France and Lebanon, dentists with more than 10 years of professional experience looked for halitosis signs more systematically than did young professionals (*p* < 0.01). Interestingly, no significant differences were observed with regards to dentist’s specialization towards halitosis.

FD and LD with a professional experience greater than 5 years were also more prone to refer patients to dentists specialized in the management of halitosis than those with less than 5 years of professional experience (*p* < 0.05). No significant differences were observed for the referral to an ENT specialist.

Similarly, dentists with professional experience of more than 20 years attended more continuous education courses focusing on halitosis than did dentists with less experience (*p* = 0.05). Accordingly, those who attended such professional education programs treated more patients for halitosis (*p* < 0.001) and assessed halitosis more systematically (*p* < 0.05). Furthermore, they often used specific diagnostic tools such as volatile sulfur compounds measurements (*p* < 0.05).

## 4. Discussion

Halitosis is a complex health problem with potentially important social consequences. As this issue is often neglected by dentists, it has increasingly become a reason for consultation. In this study, we evaluated, through a self-administered questionnaire, the knowledge and halitosis management of two dentist populations from two different cultural environments. It was observed that, even if halitosis is frequently reported or detected, appropriate diagnosis of its etiology and treatment are not systematically provided.

Halitosis impacts significantly the patient’s life as demonstrated in a recent Chinese study where it was observed that patients with genuine halitosis have worsened psychological status, including anxiety and depression [19]. Such psychological features are of importance, as social anxiety disorders may prevent the patient from seeking dental advice. Unfortunately, this decrease in patients’ confidence compromises their ability to communicate and to seek professional advice or counselling. In this study, only a limited number of French and Lebanese dentists systematically question their patients about halitosis, and most of them feel uncomfortable discussing halitosis with their patients. This result is in accordance with studies performed in other countries such as The Netherlands [11] or Australia [20]. Interestingly, this compromised confidence associated with halitosis may be explained by a lack of dedicated training and a lack of confidence in halitosis treatment. Such difficulties could be alleviated by improving communication skills of the dentists. As halitosis could be considered a taboo disease, such as overweight or obesity, specific opportunistic and structured communication strategies should be developed [21].

In this study, the diagnosis of halitosis etiology was mainly associated with intra-oral examination including, in most cases, a full periodontal examination. Only a limited number of dentists, especially those with significant professional experience or those having followed halitosis-dedicated professional education courses, used more specific diagnostic tools such as volatile sulfur compounds monitoring or the organoleptic method [22]. This observation is in accordance with other studies reporting anamnesis as the main diagnostic tool used by dentists or hygienists [8]. The organoleptic method is considered the gold standard; however, it should be performed by a trained professional in order to be effective [23]. As self-reported halitosis measures can improve screening strategies, the systematic use of self-administered questionnaires may be helpful, even for untrained dentists [23]. The use of such tools may be restricted due to lack of training and also because VSC measurement instruments are not available in most private practice offices.

In France and Lebanon, a large majority of dentists indicate that they have already treated halitosis. However, only oral hygiene instructions and treatment of periodontal diseases and caries were mainly performed. Notably, periodontitis has been positively associated to halitosis, especially in cases of severe periodontitis [3,24], as periodontal pathogens present in subgingival biofilms are able to degrade sulfur-containing substrates contributing to the release of volatile sulfur compounds [25] and dental cavities are potential food retention areas. Therefore, the proposed treatments are of high importance. It is noteworthy that some studies showed that the level of oral hygiene, estimated by the level of plaque, was not correlated with halitosis [6,10]. Therefore, a combination of effective hygiene procedures (toothbrushing, interdental brushing, tongue cleaning) with professional debridement is necessary. Interestingly, the prescription of mouthwashes, specific toothpastes, or tongue scrapers was not performed systematically by either dentist population. In this study, 51.9% of FD and 45.1% of LD consider the use of a tongue scraper to treat halitosis. These percentages are lower than those observed in other dentist populations such as Swiss, German, and Dutch [7,11]. These results could be explained by the lack of halitosis-related education, as observed for FD and LD, and also due to the unclear conclusions and recommendations regarding the intervention protocols for the efficient treatment of halitosis as shown in a recent systematic review [26]. 

In this context, the halitosis-related knowledge of FD and LD was not up to date. For instance, the extra-oral etiology of halitosis was over-estimated by a considerable proportion of FD and LD. Extra-oral halitosis can be associated with upper or lower respiratory tract infections, blood-borne disorders, or be a manifestation of some underlying serious disease. However, such types of halitosis represent only 5–10% of all cases [27]. Interestingly, in surveys of student populations, dentists and gastroenterology specialists were the most cited professionals qualified to treat halitosis, as observed in our study [14,28].

In this study, the comparison of these two populations of dentists allowed us to evaluate the social and cultural influence on halitosis management. It is noteworthy to consider the similarities and influence of France on the Lebanese education system as, for instance, the degree structure in the higher education system follows that of France’s system, and French is an official language of instruction [29]. Interestingly, several differences were observed between FD and LD. Indeed, LD were more prone to investigate the patient’s eating habits than were FD, this may be due to the differences in terms of diet habits, as the presence of garlic and spices in the Lebanese food is more prominent than in French cuisine [30]. However, such assessment should be considered with caution as no specific survey on the eating habits of both populations was performed in this study. This different take on the subject highlights the fact that in today’s multicultural society, the practitioner must be aware of the possible impact of the patient’s culture on the condition, and also adapt the solutions accordingly [31]. This study presents limitations mainly related to the use of emailing to send questionnaires. Email addresses of dentists were retrieved from professional organizations, however, only a restricted number of email addresses were collected. Nevertheless, such a method generally leads to a low rate of response. Therefore, it can be assumed that responders may have been the most interested in halitosis management or the most trained and, consequently, the knowledge of halitosis could be lower in the general population of dentists. It should also be mentioned that other parameters such as region/area of professional activity may also influence the response rate to the questionnaire. Unfortunately, data related to this parameter were not collected. However, the number of dentists that responded to this survey is in the same range as in studies performed in other countries such as Switzerland or the Netherlands [7,8,11]. However, further research should be conducted to evaluate knowledge and management of halitosis through clinically-orientated research protocols.

## 5. Conclusions

In France and Lebanon, halitosis management remains challenging for dentists. Interestingly, it seems that the efficient management of patients with halitosis by dentists is compromised by their lack of specific knowledge and training pertaining to halitosis. Therefore, there is a need to develop and promote dedicated education about halitosis at both graduate and professional education levels.

## Figures and Tables

**Table 1:** Demographic characteristics of French dentists (FD) and Lebanese dentists (LD). *p*-Value corresponds to statistical significance between FD and LD.

	France *n* (%)	Lebanon *n* (%)	*p*-Value
Gender			>0.05
Male	86 (55.1)	141 (54.9)	
Female	70 (44.9)	116 (45.1)	
Age (years old)			<0.01
<30	16 (10.3)	66 (25.7)	
30–39	36 (23.1)	60 (23.3)	
40–49	27 (17.3)	51 (18.8)	
50–59	49 (31.4)	57 (22.2)	
>59	28 (17.9)	23 (8.9)	
Professional experience (years)			<0.01
<5	18 (11.5)	60 (23.3)	
5–9	20 (12.8)	38 (14.8)	
10–19	26 (16.7)	61 (23.7)	
≥20	92 (59)	98 (38.1)	
Type of practice			<0.01
Private practice	110 (70.5)	170 (66.1)	
Dental hospital	11 (7.1)	2 (0.78)	
Dental hospital and private practice	35 (22.4)	85 (33.1)	
Dental specialization			<0.05
General practice	115 (73.7)	108 (42)	
Specialized in periodontology/implantology	44 (28.2)	28 (10.9)	
Specialized in oral surgery	16 (10.3)	37 (14.4)	
Specialized in endodontics	1 (0.6)	22 (8.6)	
Specialized in orthodontics	8 (5.1)	32 (12.5)	
Specialized in pediatric dentistry	8 (5.1)	45 (17.5)	
Specialized in prosthodontics	-	46 (17.9)	
Professional education			<0.01
Once per year	20 (12.8)	54 (21)	
Twice per year	23 (14.7)	57 (22.2)	
More than twice per year	113 (72.4)	146 (56.8)	

**Table 2:** Consideration of halitosis during dental consultation. Difference between LD and FD when adjusted for gender, age, and professional experience. A *p* < 0.05 was considered significant (OR: odds ratio, CI: confidence interval).

	France *n* (%)	Lebanon *n* (%)	OR (95% CI; *p*-Value)
Have you encountered patients or people from your surroundings suffering from lack of treatment of their halitosis?			
Yes, frequently	58 (37.2)	126 (49)	4.68 (1.42–15.44; *p* = 0.01)
Yes, rarely	84 (53.8)	124 (48.2)	2.64 (0.81–8.56; *p* = 0.11)
No, never	14 (9)	7 (2.7)	Reference
Do you receive patients consulting to treat their halitosis?			
No	28 (17.9)	50 (19.5)	Reference
Yes, less than 10 per year	92 (59)	152 (59.1)	1.34 (0.72–2.50; *p* = 0.35)
Yes, more than 10 per year	36 (23.1)	55 (21.4)	0.44 (0.18–1.05; *p* = 0.06)
Do you pay attention to halitosis in your daily practice?			
Yes, systematically	95(61)	169 (65.8)	2.88 (0.47–17.54; *p* = 0.25)
Yes, from time to time	56 (36)	82 (31.9)	2.19 (0.35–13.54; *p* = 0.40)
No, never	5 (3)	6 (2.3)	Reference
Do you ask your patients about their breath odor in your daily practice?			
Yes, systematically	25 (16)	34 (13.2)	1.49 (0.54–4.11; *p* = 0.44)
Yes, only if the patient brings the subject up	58 (37.2)	92 (35.8)	1.73 (0.72–4.15; *p* = 0.22)
Yes, only if I detect a problem of halitosis	54 (34.6)	106 (41.2)	2.04 (0.84–4.95; *p* = 0.11)
No, never	19 (12.2)	25 (9.7)	Reference
If never, why not?			
I’m afraid I will make the patient feel uncomfortable	13 (68.4)	14 (56)	Reference
This problem doesn’t fall within my practice/specialty	2 (10.5)	10 (40)	4.23 (0.41–43.59; *p* = 0.23)
I don’t’ have time for this	1 (5.3)	1 (4)	1.51 (0.08–27.97; *p* = 0.78)
Other	1 (5.3)	1 (4)	1.78 (0.09–38.19; *p* = 0.71)
A patient comes for a checkup and you detect halitosis, how do you react?			
I inform him about halitosis, and suggest he could receive a treatment	95 (61)	189 (73.5)	3.47 (1.72–7.01; *p* = 0.01)
I don’t tell him because it is embarrassing	13 (8.3)	33 (12.8)	3.16 (1.15–8.66; *p* = 0.03)
I don’t tell him because I don’t know how to help him	9 (5.7)	8 (3.1)	0.63 (0.12–3.38; *p* = 0.59)
None of the above	39 (25)	27 (10.5)	Reference

**Table 3:** Diagnosis and management of halitosis. Difference between LD and FD when adjusted for gender, age, and professional experience. A *p* < 0.05 was considered significant. (OR: odds ratio, CI: confidence interval).

	France *n* (%)	Lebanon *n* (%)	OR (95% CI; *p*-Value)
Have you ever treated patients for halitosis?			
Yes	123 (78.8)	177 (68.9)	0.90 (0.49–1.63; *p* = 0.72)
No	33 (21.2)	80 (31.1)	Reference
If yes, how many patients have you treated in the last six months?			
Less than 5	82 (66.7)	139 (78.5)	1.92 (0.58–6.40; *p* = 0.29)
Between 5 and 15	29 (23.6)	27 (15.3)	1.15 (0.30–4.44; *p* = 0.83)
More than 15	12 (9.8)	11 (6.2)	Reference
When you decide to treat halitosis, which questions do you ask in order to orient your etiological diagnosis?			
What are your oral hygiene habits?	112 (71.8)	146 (56.8)	0.35 (0.14–0.84; *p* = 0.02)
What is the history/anamnesis of your halitosis?	89 (57.1)	137 (53.3)	1.09 (0.56–2.10; *p* = 0.81)
Do you suffer from a systemic disease (diabetes, kidney failure…)	101 (64.7)	141 (54.8)	0.98 (0.47–2.04; *p* = 0.96)
Have you consulted other health professionals (ENT specialist, gastroenterologist)	78 (50)	119 (46.3)	1.05 (0.58–1.91; *p* = 0.87)
Other	15 (9.6)	26 (10.1)	1.35 (0.57–3.17; *p* = 0.49)
When you decide to treat halitosis which specific diagnostic tools do you use?			
Full-mouth periodontal charting	101 (64.7)	139 (54.1)	1.72 (0.78–3.80; *p* = 0.18)
Organoleptic score	6 (3.8)	12 (4.7)	2.25 (0.63–8.10; *p* = 0.21)
Measurement of volatile sulfur compounds (Halimeter^®^, OralChroma^®^)	3 (1.9)	16 (6.2)	3.50 (0.61–20.23; *p* = 0.16)
Precise investigation of the patient’s eating habits/diet	53 (34)	112 (43.8)	2.22 (1.24–3.98; *p* = 0.007)
Other	20 (12.8)	23 (8.9)	0.62 (0.26–1.48; *p* = 0.28)
If your patient suffers from halitosis of intra-oral etiology, how do you proceed to treat it?			
Give oral hygiene instructions	116 (74.4)	166 (64.6)	0.83 (0.25–2.70; *p* = 0.76)
Caries and periodontal treatment	114 (73.1)	163 (63.4)	1.83 (0.54–6.17; *p* = 0.33)
Prescription of toothpaste	55 (35.3)	74 (28.8)	0.94 (0.52–1.71; *p* = 0.84)
Prescription of mouthwash for bad breath	83 (53.2)	134 (52.1)	1.81 (0.94–3.50; *p* = 0.08)
Prescription of tongue scraper	81 (51.9)	116 (45.1)	0.94 (0.51–1.71; *p* = 0.83)
Other	7 (4.5)	10 (3.9)	1.26 (0.36–4.37; *p* = 0.71)
What do you think about your treatment and its outcomes?			
It is efficient (suppression of halitosis in most of the cases)	62 (39.7)	73 (28.4)	0.86 (0.43–1.70; *p* = 0.66)
I need new products in some cases	20 (12.8)	53 (20.6)	1.58 (0.72–3.48; *p* = 0.25)
I think I could do better	41 (26.3)	51 (19.8)	Reference

**Table 4:** Referral and need for education. Difference between LD and FD when adjusted for gender, age, and professional experience. A *p* < 0.05 was considered significant. (OR: odds ratio, CI: confidence interval).

	France *n* (%)	Lebanon *n* (%)	OR (95% CI; *p*-Value)
Have you ever referred patients to get treated for halitosis?			
Yes	42 (26.9)	98 (38.1)	2.02 (1.17–3.49; *p* = 0.01)
No	114 (73.1)	159 (61.9)	Reference
If yes, to whom do you refer them to?			
A dentist specialized in the treatment of halitosis	4 (9.5)	23 (23.5)	2.25 (0.44–11.43), *p* = 0,33)
An odontology department specialized in the treatment of halitosis	9 (21.4)	13 (13.3)	0.44 (0.11–1.79; *p* = 0.25)
An ENT specialist	25 (59.5)	54 (55.1)	0.82 (0.31–2.19; *p* = 0.70)
A general practitioner	12 (28.6)	37 (37.8)	2.52 (0.88–7.19; *p* = 0.08)
A psychologist	0 (0)	1 (1)	1.83 (0.05–61.87; *p* = 0.74)
If not, for which reasons?			
I don’t usually treat halitosis	10 (6.4)	18 (11.3)	1.99 (0.72–2.50; *p* = 0.19)
These treatments are not taken covered by the insurance companies	4 (2.6)	4 (2.5)	1.02 (0.14–7.25; *p* = 0.98)
I don’t know any specialist or institution that treats this problem	66 (42.3)	88 (55.3)	1.70 (0.94–3.08; *p* = 0.08)
I know how to treat halitosis myself	42 (26.9)	53 (20.6)	1.60 (0.84–3.03; *p* = 0.15)
Other reasons	48 (30.8)	33 (20.8)	0.53 (0.26–1.08; *p* = 0.08)
If you don’t actually treat halitosis, are you willing to do it?			
Yes	30 (90.9)	57(71.3)	0.26 (0.02–4.19; *p* = 0.34)
No	3 (9.1)	23 (28.8)	Reference
If yes what could help you do it?			
Coverage of the treatment’s cost by the national health organization/private health insurances	34 (21.8)	18 (7)	0.21 (0.09–0.49; *p* < 0.001)
Ongoing institutional education (University…)	44 (28.2)	87 (33.8)	1.12 (0.63–1.97; *p* = 0.70)
Ongoing professional education (Congresses, training sessions…)	78 (50)	153 (59.64)	1.43 (0.81–2.52; *p* = 0.22)
Publications (Scientific journals, books…)	60 (38.5)	154 (59.9)	1.94 (1.12–3.36; *p* = 0.02)
Dental sale representatives	16 (10.3)	55 (21.4)	2.79 (1.33–5.88; *p* = 0.007)
New products	47 (30.1)	119 (46.3)	2.57 (1.47–4.5; *p* = 0.001)
I don’t know	20 (12.8)	14 (5.4)	0.15 (0.048–0.46; *p* = 0.001)

**Table 5:** Halitosis related knowledge of FD and LD. Difference between LD and FD when adjusted for gender, age, and professional experience. A *p* < 0.05 was considered significant. (OR: odds ratio, CI: confidence interval).

	France *n* (%)	Lebanon *n* (%)	OR (95% CI; *p*-Value)
How would you qualify your education about halitosis at your dental school?			
Excellent	0 (0)	7 (2.7)	0.52 (0.37–0.67; *p* < 0.001)
Sufficient	18 (11.5)	76 (29.6)	
Insufficient	75 (48.1)	148 (57.6)	
Non-existent	55 (35.3)	21 (8.2)	
I don’t know	8 (5.1)	5 (1.9)	
Have you participated in continuous educational courses aimed at halitosis?			
Yes	40 (25.6)	53 (20.6)	0.93 (0.52–1.64; *p* = 0.806)
No	116 (74.4)	196 (76.3)	
If yes, why?			
This subject interests me	21 (52.5)	29 (54.7)	0.72 (0.17–3.03; *p* = 0.655) 2.74
Some of my patients suffer from halitosis	14 (35)	35 (66)	(0.79–9.51; *p* = 0.112)
Halitosis is a subject that is brought up in the field of my specialty (Periodontology…)	30 (75)	23 (43.4)	0.34 (0.10–1.21; *p* = 0.096)
I have had the opportunity	7 (17.5)	16 (30.2)	1.13 (0.22–5.74; *p* = 0.881)
I don’t know	0 (0)	2 (3.8)	
If no, why?			
Lack of time	7 (6)	18 (9.1)	1.49 (0.45–4.98; *p* = 0.511)
I didn’t find an appropriate educational course	54 (46.6)	123 (62.8)	2.64 (1.42–4.91; *p* = 0.002)
This subject isn’t of high priority	48 (41.4)	65 (33.2)	0.59 (0.32–1.10; *p* = 0.096)
It is already planned	8 (7)	8 (4.1)	0.58 (0.15–2.21; *p* = 0.427)
I don’t know	14 (12.1)	22 (11.3)	0.45 (0.16–1.25; *p* = 0.125)
Do you think that dentists are well-trained to be able to diagnose and treat halitosis?			
Yes	14 (9)	35 (13.6)	2.56 (1.12–5.84; *p* = 0.025)
No	88 (56.4)	123 (47.9)	Reference
I don’t know	54 (34.6)	99 (38.5)	
In your opinion,			
Halitosis is irreversible	2 (1.3)	7 (2.7)	1.61 (0.24–10.94; *p* = 0.63)
Halitosis can be physiological	57 (36.5)	132 (51.3)	1.65 (1.00–2.72; *p* = 0.09)
Halitosis reflects a pathology of oral or general etiology	133 (85.3)	203 (78.9)	0.86 (0.45–1.67; *p* = 0.66)
Halitosis is always associated with bad oral hygiene	20 (12.8)	58 (22.6)	3.12 (1.62–6.01; *p* < 0.001)
Halitosis is purely subjective	3 (1.9)	9 (3.5)	0.19 (0.02–1.96; *p* = 0.16)
Bad taste is always associated to halitosis	10 (6.4)	27 (10.5)	1.84 (0.74–4.58; *p* = 0.19)
Morning halitosis disappears with brushing or breakfast	53 (34)	136 (52.9)	2.61 (1.55–4.38; *p* < 0.001)
In cases of halitophobia it is better to refer the patient to a psychologist	40 (25.6)	68 (25.5)	1.10 (0.63–1.93; *p* = 0.74)
Organoleptic measurements are made with saliva samples	8 (5.1)	26 (10.1)	2.31 (0.88–6.08; *p* = 0.09)
The patient always detects his own bad breath+	5 (3.2)	35 (13.6)	5.78 (1.90–17.51; *p* = 0.002)
Halitosis is caused by volatile sulfur compounds+	108 (69.2)	123 (47.9)	0.60 (0.36–1.00; *p* = 0.049)
30% of halitosis are of extra-oral causes (gastric, ENT…)	95 (61)	190 (73.9)	1.76 (1.05–2.97; *p* = 0.034)
Prescription of antibiotics is the treatment of choice	3 (1.9)	2 (0.8)	0.17 (0.01–2.83; *p* = 0.22)
Mouthwash rinses are efficient in the treatment of halitosis	58 (37.2)	144 (56)	1.83 (1.10–3.04; *p* = 0.02)
How did you build your knowledge around this subject?			
During years of dental school	58 (37.2)	150 (58.4)	1.69 (1.00–2.87; *p* = 0.05)
During one session of an ongoing educational course (congress, seminar…)	46 (29.5)	93 (36.2)	1.54 (0.90–2.64; *p* = 0.12)
Colleague	15 (9.6)	25 (9.7)	0.85 (0.37–1.95; *p* = 0.71)
Scientific journals	90 (57.7)	76 (29.6)	0.30 (0.18–0.51; *p* < 0.001)
Dental sale representative	29 (18.6)	23 (8.9)	0.45 (0.21–0.95; *p* = 0.04)
Mainstream press	14 (9)	49 (19.1)	2.41 (1.11–5.24; *p* = 0.03)
Internet	14 (9)	80 (31.1)	5.86 (2.82–12.19; *p <* 0.001)
Television show	2 (1)	3 (1.2)	0.52 (0.07–4.02; *p* = 0.53
Other	8 (3.1)	14 (5.4)	1.48 (0.53–4.17; *p* = 0.46)

## Data Availability

All data are available from authors on reasonable request.

## References

[1] Akaji E.A., Folaranmi N., Ashiwaju O. (2014). Halitosis: A review of the literature on its prevalence, impact and control. Oral Health Prev. Dent..

[2] Silva M.F., Leite F.R.M., Ferreira L.B., Pola N.M., Scannapieco F.A., Demarco F.F., Nascimento G.G. (2018). Estimated prevalence of halitosis: A systematic review and meta-regression analysis. Clin. Oral Investig..

[3] Silva M.F., Cademartori M.G., Leite F.R.M., Lopez R., Demarco F.F., Nascimento G.G. (2017). Is periodontitis associated with halitosis? A systematic review and meta-regression analysis. J. Clin. Periodontol..

[4] Seerangaiyan K., Jüch F., Winkel E.G. (2018). Tongue coating: Its characteristics and role in intra-oral halitosis and general health—A review. J. Breath Res..

[5] Murata T., Yamaga T., Iida T., Miyazaki H., Yaegaki K. (2002). Classification and examination of halitosis. Int. Dent. J..

[6] Quirynen M., Dadamio J., Velde S.V.D., De Smit M., DeKeyser C., Van Tornout M., Vandekerckhove B. (2009). Characteristics of 2000 patients who visited a halitosis clinic. J. Clin. Periodontol..

[7] Oppliger N., Roth B., Filippi A. (2014). Knowledge of halitosis among dentists and dental hygienists. Swiss Dent. J..

[8] Roth B., Oppliger N., Filippi A. (2014). Knowledge of different medical and dental professional groups in Switzerland about halitosis. Swiss Dent. J..

[9] Kapoor U., Sharma G., Juneja M., Nagpal A. (2016). Halitosis: Current concepts on etiology, diagnosis and management. Eur. J. Dent..

[10] Lu H.-X., Tang C., Chen X., Wong M., Ye W. (2013). Characteristics of patients complaining of halitosis and factors associated with halitosis. Oral Dis..

[11] Buunk-Werkhoven Y.A.B., Buls J.G., Osinga E., Bruers J.J.M. (2015). Diagnosis and treatment of patients with halitosis by dental hygienists and dentists in the Netherlands. Int. Dent. J..

[12] Rayman S., Almas K. (2008). Halitosis among racially diverse populations: An update. Int. J. Dent. Hyg..

[13] Schemel-Suárez M., Chimenos-Küstner E., Estrugo-Devesa A., Jané-Salas E., López-López J. (2017). Halitosis assessment and changes in volatile sulfur compounds after chewing gum: A study performed on dentistry students. J. Evid. Based Dent. Pract..

[14] Mubayrik A.B., Al Hamdan R., Al Hadlaq E.M., Al Bagieh H., Al Ahmed D., Jaddoh H., Demyati M., Abu Shryei R. (2017). Self-perception, knowledge, and awareness of halitosis among female university students. Clin Cosmet. Investig. Dent..

[15] Wu J., Cannon R.D., Ji P., Farella M., Mei L. (2020). Halitosis: Prevalence, risk factors, sources, measurement and treatment—A review of the literature. Aust. Dent. J..

[16] Frexinos J., Denism P., Allemand H., Allouche S., Los F., Bonnelye G. (1998). Etude descriptive des symptômes fonctionnels digestifs dans la population générale française [Descriptive study of digestive functional symptoms in the French general population]. Gastroenterol. Clin. Biol..

[17] Eldarrat A., Alkhabuli J., Malik A. (2008). The prevalence of self-reported halitosis and oral hygiene practices among libyan students and office workers. Libyan J. Med..

[18] Gelman A.B., Jakulin A., Pittau M.G., Su Y.-S. (2008). A weakly informative default prior distribution for logistic and other regression models. Ann. Appl. Stat..

[19] He M., Lu H., Cao J., Zhang Y., Wong M.C.M., Fan J., Ye W. (2020). Psychological characteristics of Chinese patients with genuine halitosis. Oral Dis..

[20] Lau P., Meethal C., Middleton M., Clark M., Darby I. (2019). “Say Ahhh”: What do dentists, general medical practitioners and community pharmacists do about halitosis?. Int. Dent. J..

[21] Gray L., Stubbe M., Macdonald L., Tester R., Hilder J., Dowell A. (2018). A taboo topic? How General Practitioners talk about overweight and obesity in New Zealand. J. Prim. Heal. Care.

[22] Nakhleh M.K., Quatredeniers M., Haick H. (2018). Detection of halitosis in breath: Between the past, present, and future. Oral Dis..

[23] Faria S.F.S., Costa F.O., Silveira J.O., Cyrino R.M., Cota L.O.M. (2020). Self-reported halitosis in a sample of Brazilians: Prevalence, associated risk predictors and accuracy estimates with clinical diagnosis. J. Clin. Periodontol..

[24] Aimetti M., Perotto S., Castiglione A., Ercoli E., Romano F. (2015). Prevalence estimation of halitosis and its association with oral health-related parameters in an adult population of a city in North Italy. J. Clin. Periodontol..

[25] Nakano Y., Yoshimura M., Koga T. (2002). Methyl mercaptan production by periodontal bacteria. Int. Dent. J..

[26] Nagraj S.K., Eachempati P., Uma E., Singh V.P., Ismail N.M., Varghese E. (2019). Interventions for managing halitosis. Cochrane Database Syst. Rev..

[27] Tangerman A., Winkel E.G. (2010). Extra-oral halitosis: An overview. J. Breath Res..

[28] Nunes J.C., Sahuquillo Á.M., Cameira M.J., Marques H.D. (2011). Halitosis: Are dentists being prepared for this chal-lenge? A questionnaire survey in a dental school. Rev. Port. Estomatol. Med. Dentária Cir. Maxilofac..

[29] Loo B., Magaziner J. Education in Lebanon. World Education News & Reviews [Internet]. https://wenr.wes.org/2017/05/education-in-lebanon.

[30] Hwalla N., El Khoury D.T.D., De Meester F., Watson R.R. (2008). Lebanese traditional diets and health effects. Wild-Type Food in Health Promotion and Disease Prevention: The Columbus Concept.

[31] Putsch R.W., Joyce M., Walker H.K., Hall W.D., Hurst J.W. (1990). Dealing with patients from other cultures. Clinical Methods: The History, Physical, and Laboratory Examinations.

